# Nobiletin as a Geroprotective Flavonoid: Mechanisms of Action, Therapeutic Potential, and Challenges of Bioavailability

**DOI:** 10.3390/antiox15091129

**Published:** 2026-09-07

**Authors:** Szymon Sip, Anna Gościniak, Niklas Hoede, Min Hein Htike, Hnin Yuzana Khin, Wei-Wei Lai, Hein Htet Naing, Agnieszka Szopa, Judyta Cielecka-Piontek

**Affiliations:** 1 Department of Pharmacognosy and Biomaterials, Poznan University of Medical Sciences, Rokietnicka 3, 60-806 Poznan, Poland; agosciniak@ump.edu.pl; 2 Doctoral School, Poznan University of Medical Sciences, Bukowska Str. 70, 60-812 Poznan, Poland; 90671@student.ump.edu.pl (N.H.); 90637@student.ump.edu.pl (M.H.H.); 90625@student.ump.edu.pl (H.Y.K.); 90619@student.ump.edu.pl (W.-W.L.); 90651@student.ump.edu.pl (H.H.N.); 3 Department of Medicinal Plant and Mushroom Biotechnology, Faculty of Pharmacy, Jagiellonian University Medical College, Medyczna 9, 30-688 Kraków, Poland; a.szopa@uj.edu.pl

**Keywords:** nobiletin, geroprotector, geroscience, cellular senescence, inflammaging, mitophagy, circadian rhythm, healthy ageing, mitochondrial dysfunction, bioavailability

## Abstract

Flavonoids are a diverse group of naturally occurring polyphenolic compounds found in plant-based foods and are known for their broad-spectrum biological activities. Among them, nobiletin, a polymethoxylated flavone derived from citrus peels, has gained attention for its multifaceted role in promoting healthy ageing and mitigating age-related diseases. This review explores the chemical properties, mechanisms of action, therapeutic potential, and challenges related to nobiletin. The focus is placed on its antioxidant, anti-inflammatory, neuroprotective, cardioprotective, and metabolic regulatory effects, as well as its capacity to enhance mitophagy, modulate circadian rhythms, and prevent ferroptosis. Despite promising preclinical results across various models, including in vitro cell lines, *C. elegans*, and mice, the clinical translation of nobiletin is hindered by poor oral bioavailability, complex extraction, and limited human data. Novel delivery systems, such as nanoemulsions and phospholipid-based carriers, offer potential solutions to enhance its bioavailability. As research advances, nobiletin emerges as a promising candidate for geroprotective therapy, warranting further investigation through well-designed clinical trials to extend healthspan and prevent age-associated pathologies.

## 1. Introduction

### 1.1. Overview of Flavonoids and Their Effects on Health and Aging

Flavonoids constitute a structurally diverse group of naturally occurring polyphenolic compounds widely distributed in fruits, vegetables, tea, cocoa, and other plant-derived foods. Based on their chemical structure, they are classified into several major subclasses: flavones, flavonols, flavanones, flavan-3-ols, anthocyanins, and isoflavones [1]. Citrus-derived flavonoids have attracted particular scientific interest due to their broad spectrum of biological activities and potential to prevent chronic diseases and promote healthy ageing. Among these compounds, nobiletin, a polymethoxylated flavone predominantly present in citrus peels, has emerged as a particularly promising bioactive molecule.

A substantial body of experimental evidence indicates that flavonoids help maintain cellular redox homeostasis. Their antioxidant effects may involve direct scavenging of reactive oxygen species (ROS), chelation of redox-active metal ions, and modulation of endogenous antioxidant defence systems [2]. In particular, flavonoids have been reported to influence the activity or expression of enzymes such as superoxide dismutase (SOD), catalase, and glutathione peroxidase (GPx), thereby limiting oxidative damage to lipids, proteins, and nucleic acids [3]. Because persistent oxidative stress is closely associated with cellular senescence, mitochondrial dysfunction, and the development of age-related disorders, the antioxidant activity of flavonoids may help protect against cardiovascular, metabolic, neurodegenerative, and neoplastic diseases [4,5].

In addition to their effects on redox balance, flavonoids exhibit pronounced anti-inflammatory properties. Flavonoids may attenuate age-related chronic inflammation by modulating NF-κB and MAPK signalling and reducing the production of pro-inflammatory mediators [6,7]. Through these mechanisms, they may attenuate systemic inflammation and support tissue homeostasis. These effects have been attributed, at least in part, to increased nitric oxide bioavailability, reduced oxidative inactivation of nitric oxide, inhibition of platelet aggregation, and attenuation of low-density lipoprotein oxidation [8,9].

More evidence further suggests that flavonoids may support neuronal function and cognitive health. Some of these effects are associated with modulation of signalling pathways involving brain-derived neurotrophic factor (BDNF), which plays an essential role in neuronal survival, synaptic remodelling, learning, and memory [10,11,12]. These effects involve several intracellular pathways, including AMP-activated protein kinase (AMPK) and peroxisome proliferator-activated receptor gamma (PPARγ), both of which regulate energy balance, adipogenesis, lipid storage, and glucose metabolism [13,14]. Observational evidence has linked flavonoid-rich dietary patterns with a lower incidence of metabolic syndrome and type 2 diabetes mellitus. However, such associations should be interpreted cautiously because they may also reflect the broader health benefits of diets rich in fruits, vegetables, and other plant-derived foods.

### 1.2. Chemical and Physicochemical Properties of Nobiletin

**Figure 1:**
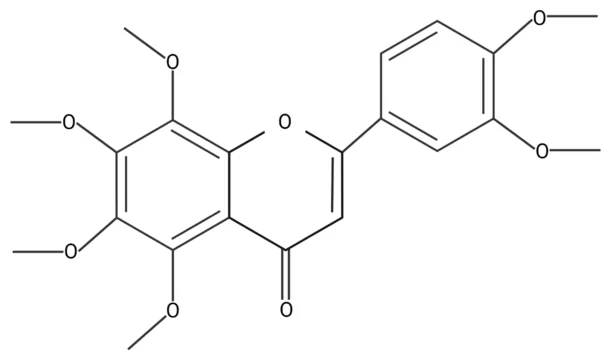
Chemical structure of nobiletin.

Nobiletin is a naturally occurring polymethoxylated flavone (PMF) predominantly localised in the flavedo (outer peel) of citrus fruits, including *Citrus reticulata* (mandarin), *Citrus tangerina* (tangerine), and *Citrus paradisi* (grapefruit). Among citrus species, tangerines are considered one of the richest natural sources of this compound. The high concentration of nobiletin in citrus peels has attracted considerable interest in utilising citrus processing by-products as sustainable sources of bioactive phytochemicals for pharmaceutical and nutraceutical applications. Chemically, nobiletin is identified as 5,6,7,8,3′,4′-hexamethoxyflavone (Figure 1), belonging to the flavone subclass of flavonoids. Its molecular structure consists of the characteristic C6–C3–C6 flavonoid backbone substituted with six methoxy (-OCH_3_) groups, which distinguish it from hydroxylated flavonoids such as quercetin or luteolin. Extensive methylation substantially increases its hydrophobicity and chemical stability while decreasing susceptibility to oxidative degradation and phase II metabolism. Consequently, nobiletin exhibits greater metabolic stability than many other naturally occurring flavonoids [15,16].

From a physicochemical perspective, nobiletin is a yellow crystalline compound with a molecular formula of C_21_H_22_O_8_ and a molecular weight of approximately 402.39 g/mol. Owing to the presence of multiple methoxy substituents, it exhibits pronounced lipophilicity, reflected by a relatively high octanol/water partition coefficient (logP) [17]. As a result, nobiletin demonstrates very low aqueous solubility but readily dissolves in organic solvents such as ethanol, methanol, dimethyl sulfoxide (DMSO), acetone, and chloroform. These physicochemical characteristics facilitate passive diffusion across biological membranes, including the blood–brain barrier, but simultaneously limit its dissolution in gastrointestinal fluids following oral administration. Therefore, despite favourable membrane permeability, its oral bioavailability remains relatively low because dissolution becomes the rate-limiting step in absorption.

Nobiletin is particularly relevant to geroscience because its biological activity extends beyond general antioxidant and anti-inflammatory effects. Experimental evidence indicates that it may influence several interconnected processes involved in ageing, including mitochondrial quality control [18,19], circadian regulation and metabolic homeostasis [19,20,21], and age-related tissue dysfunction [18]. Nobiletin has also been shown to extend lifespan and increase stress resistance in *Caenorhabditis elegans* [17]. These findings distinguish nobiletin from the broader epidemiological evidence concerning dietary flavonoids and support its investigation as a potential multi-target geroprotective compound.

Therefore, this narrative review aims to critically evaluate the current evidence on the geroprotective potential of nobiletin, with particular emphasis on its effects on oxidative stress, mitochondrial dysfunction, chronic inflammation, cellular senescence, and circadian regulation. It also summarises the available preclinical and clinical evidence, examines the pharmacokinetic and bioavailability limitations that may hinder its therapeutic application, and identifies key research gaps and priorities for its future clinical translation.

## 2. Literature Search and Selection Criteria

This narrative review summarises current knowledge on the chemical properties, biological activities, therapeutic potential, bioavailability, and future perspectives of nobiletin in the context of healthy ageing and age-related diseases. The literature search was conducted using electronic databases, including PubMed, Scopus, Web of Science, and Google Scholar. The search strategy included combinations of the following keywords: “nobiletin,” “polymethoxylated flavones,” “citrus flavonoids,” “aging,” “longevity,” “healthy aging,” “oxidative stress,” “inflammation,” “mitophagy,” “mitochondrial function,” “circadian rhythm,” “metabolic disorders,” “neuroprotection,” “cardioprotection,” “bioavailability,” “pharmacokinetics,” and “drug delivery systems.”

The review identifies the main evidence gaps and priorities for future research. This narrative review was based on searches of PubMed, Scopus, and Web of Science for publications from January 2020 to July 2026. Earlier publications from 2006 to 2019 were also included when they provided relevant foundational or mechanistic evidence. Original in vitro, in vivo, and human studies were considered. Only publications written in English were included. No printed-only materials were consulted; all sources used in this review were retrieved from electronic databases or journal websites. Since this article was designed as a narrative review, no formal systematic review protocol, risk-of-bias assessment, or meta-analysis was performed. Nevertheless, the literature selection aimed to present a balanced overview of the available evidence, including both the promising biological effects of nobiletin and the limitations that currently restrict its clinical translation.

## 3. Mechanism of Action

### 3.1. Role of Nobiletin in Enhancing Cellular Longevity Through the Regulation of Mitophagy

Mitochondria are highly dynamic organelles that play a central role in cellular energy metabolism and are essential for maintaining cellular homeostasis [22]. Besides being the primary site of ATP production through oxidative phosphorylation, mitochondria regulate numerous physiological processes, including calcium homeostasis, apoptosis, ROS signalling, and innate immune responses [23]. Because of these diverse functions, mitochondrial integrity is fundamental for normal cellular activity. In contrast, mitochondrial dysfunction is recognised as a hallmark of ageing and a major contributor to the development of age-related diseases, including neurodegenerative disorders, cardiovascular diseases, metabolic syndrome, and cancer [24].

During oxidative phosphorylation, mitochondria continuously generate ROS as unavoidable by-products of electron transport. Under physiological conditions, ROS participate in intracellular signalling and are efficiently neutralised by endogenous antioxidant systems [25]. However, during ageing or under conditions of metabolic stress, excessive ROS production overwhelms antioxidant defences, leading to oxidative damage to mitochondrial DNA (mtDNA), proteins, and membrane lipids [26]. Such damage impairs mitochondrial respiration, reduces ATP production, and promotes the accumulation of dysfunctional mitochondria, thereby initiating a vicious cycle of increased ROS production and progressive cellular dysfunction. Consequently, mitochondrial quality maintenance is considered a critical determinant of cellular longevity and organismal healthspan [27].

To preserve mitochondrial homeostasis, eukaryotic cells employ sophisticated quality-control mechanisms, including mitochondrial biogenesis, mitochondrial dynamics (fusion and fission), and mitophagy [28]. Mitophagy is a specialised form of selective autophagy that identifies and degrades damaged or dysfunctional mitochondria before they can trigger excessive oxidative stress or apoptosis [29]. Efficient mitophagy is therefore indispensable for maintaining mitochondrial function and preventing the accumulation of defective organelles that contribute to cellular senescence and chronic inflammation. Impaired mitophagy has been increasingly implicated in ageing and in the pathogenesis of numerous disorders, including Alzheimer’s disease, Parkinson’s disease, sarcopenia, cardiovascular disease, and type 2 diabetes mellitus [30,31].

**Figure 2:**
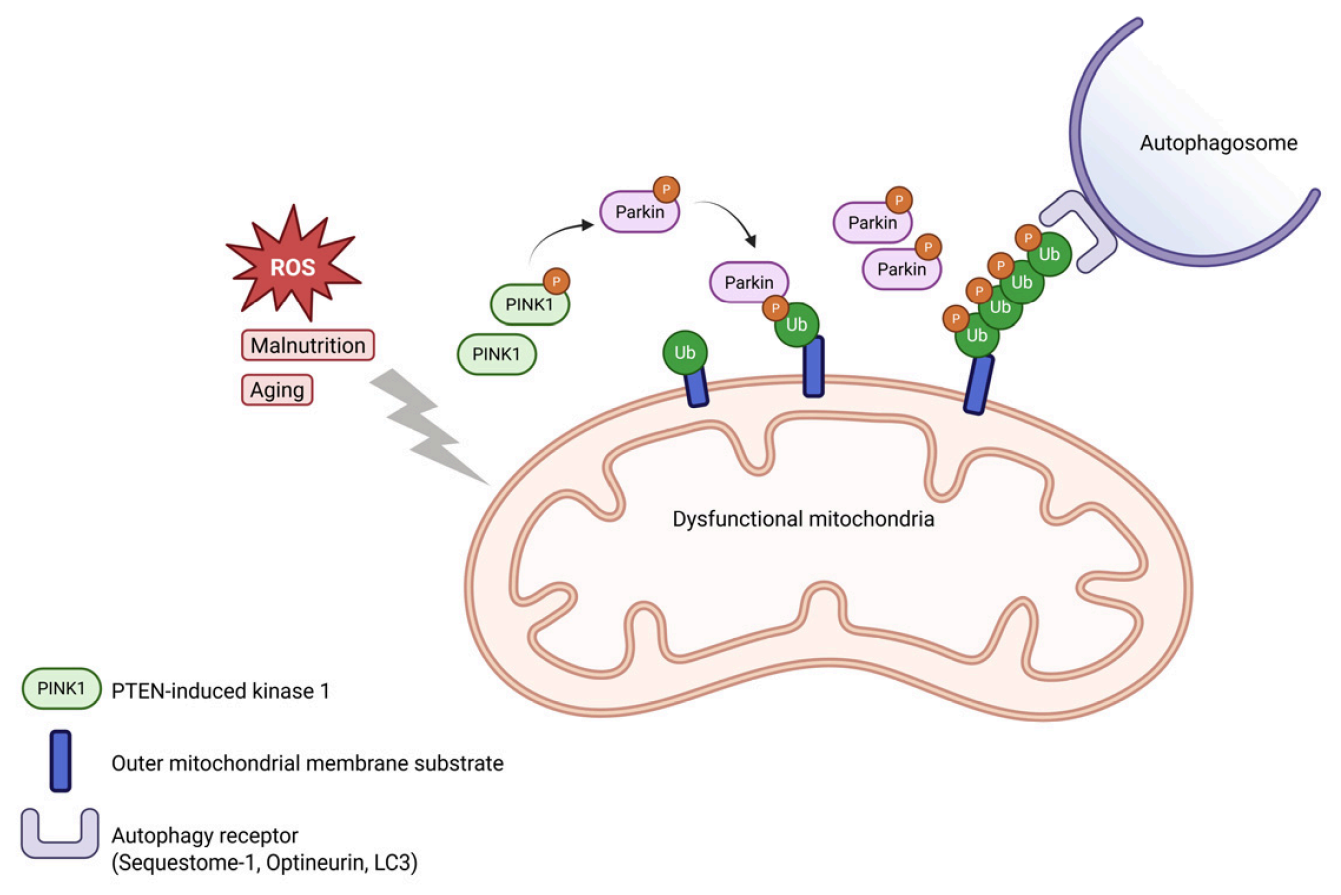
Mitophagy through the PINK1-Parkin pathway. Abbreviations: LC3, microtubule-associated protein 1 light chain 3; P, phosphorylation; PINK1, PTEN-induced kinase 1; ROS, reactive oxygen species; Ub, ubiquitin.

The best-characterised mechanism regulating mitophagy is the ubiquitin-dependent PINK1–Parkin signalling pathway [32]. Under physiological conditions, PTEN-induced kinase 1 (PINK1) is continuously imported into healthy mitochondria and rapidly degraded following translocation across the mitochondrial membranes. In contrast, mitochondrial depolarisation caused by oxidative damage or metabolic stress prevents PINK1 from being imported into the mitochondrial matrix, resulting in its accumulation on the outer mitochondrial membrane (OMM). Stabilised PINK1 undergoes autophosphorylation and subsequently phosphorylates both ubiquitin molecules and the cytosolic E3 ubiquitin ligase Parkin, thereby promoting Parkin recruitment and activation [33,34]. The principal steps involved in PINK1–Parkin-mediated mitophagy and the proposed influence of nobiletin on mitochondrial quality control are illustrated in Figure 2.

Activated Parkin ubiquitinates numerous proteins located on the outer mitochondrial membrane, including mitofusins (MFN1 and MFN2), voltage-dependent anion channels (VDAC), and mitochondrial Rho GTPases (MIRO). These ubiquitinated proteins serve as molecular signals for selective mitochondrial elimination. Polyubiquitin chains generated by Parkin are recognised by autophagy adaptor proteins, including Sequestosome-1 (p62/SQSTM1), Optineurin (OPTN), and Nuclear Dot Protein 52 (NDP52), which simultaneously bind ubiquitin and the autophagosomal membrane protein microtubule-associated protein 1 light chain 3 (LC3) [35]. This interaction facilitates the recruitment of autophagosomal membranes around damaged mitochondria, ultimately leading to the formation of mature autophagosomes. Subsequent fusion of autophagosomes with lysosomes results in enzymatic degradation of dysfunctional mitochondria and recycling of their molecular components [36]. Growing experimental evidence indicates that nobiletin positively regulates mitochondrial quality control by enhancing mitophagy through several complementary mechanisms. In both in vitro and in vivo studies, nobiletin has been shown to activate the PINK1–Parkin signalling cascade, thereby facilitating the selective removal of dysfunctional mitochondria. This effect was observed in D-galactose-induced senescent chicken ovarian granulosa cells and naturally ageing laying hens, as well as in macrophage-based atherosclerosis models and ApoE^−^/^−^ mice, where nobiletin increased the expression of PINK1/Parkin pathway-related proteins and improved mitochondrial quality control [18,37]. In addition, treatment with nobiletin significantly increases the expression of autophagy-related proteins, particularly LC3B, while simultaneously accelerating the degradation of p62, indicating enhanced autophagic flux rather than mere accumulation of autophagic vesicles. These molecular changes promote more efficient turnover of damaged mitochondria, preserve mitochondrial membrane potential, and improve respiratory function, ultimately limiting intracellular oxidative stress [18].

Beyond stimulating mitophagy, nobiletin has also been reported to improve mitochondrial function by reducing ROS generation, preserving mitochondrial membrane integrity, and enhancing ATP production. Improved mitochondrial quality contributes to decreased activation of pro-inflammatory pathways and reduced apoptosis, thereby attenuating several fundamental mechanisms of biological ageing [38]. Furthermore, by maintaining mitochondrial homeostasis, nobiletin may indirectly suppress the development of the senescence-associated secretory phenotype (SASP), which is characterised by chronic production of inflammatory cytokines and contributes to tissue dysfunction during ageing [39,40]. Evidence supporting these mechanisms has recently been strengthened by studies in ageing chicken ovarian follicles, in which nobiletin significantly enhanced mitophagy, reduced oxidative stress, activated AMPK/SIRT1 signalling, and improved follicular integrity [18]. Similar observations have been reported in skeletal muscle cells and other experimental models of ageing, in which nobiletin restored mitochondrial homeostasis, increased cellular stress resistance, and delayed the onset of senescence-associated phenotypes. These findings suggest that enhancing mitochondrial quality control through mitophagy activation is one of the principal geroprotective mechanisms of nobiletin. By preserving mitochondrial function and limiting oxidative damage, nobiletin may help maintain cellular homeostasis, delay age-associated functional decline, and reduce the risk of chronic diseases associated with ageing.

### 3.2. Role of Nobiletin in Suppressing Inflammation

Chronic inflammation is recognised as one of the fundamental hallmarks of ageing and plays a central role in the pathogenesis of numerous age-related disorders, including cardiovascular disease, neurodegenerative diseases, metabolic syndrome, diabetes mellitus, and cancer. Unlike acute inflammation, which is a tightly regulated, self-limiting protective response to tissue injury or infection, chronic low-grade inflammation persists for prolonged periods and contributes to progressive tissue damage [41]. This age-associated inflammatory state, commonly referred to as inflammaging, is characterised by continuous production of pro-inflammatory mediators that disrupt tissue homeostasis and accelerate biological ageing [42]. Consequently, modulation of inflammatory signalling pathways has emerged as an important therapeutic strategy for delaying ageing and preventing chronic diseases. The inflammatory response is initiated through the sequential production of numerous inflammatory mediators, including nitric oxide (NO), prostaglandins (PGs), chemokines, and cytokines. These mediators are produced following activation of innate immune cells, including macrophages, dendritic cells, and neutrophils, in response to pathogenic microorganisms or endogenous danger signals released from damaged tissues. Recognition of pathogen-associated molecular patterns (PAMPs) and damage-associated molecular patterns (DAMPs) by pattern recognition receptors (PRRs), particularly Toll-like receptors (TLRs), activates intracellular signalling cascades that coordinate the inflammatory response. Among these pathways, the nuclear factor of kappa light chain enhancer (NF-κB) and mitogen-activated protein kinase (MAPK) signalling pathways are the principal regulators of inflammatory gene expression. Activation of the NF-κB pathway promotes phosphorylation and degradation of the inhibitory protein IκB, allowing NF-κB heterodimers, primarily composed of the p65 (RelA) and p50 subunits, to translocate into the nucleus. Once inside the nucleus, these transcription factors bind specific promoter regions and induce the expression of numerous pro-inflammatory genes encoding cytokines such as tumour necrosis factor-α (TNF-α), interleukin-1β (IL-1β), and interleukin-6 (IL-6), as well as inflammatory enzymes including inducible nitric oxide synthase (iNOS) and cyclooxygenase-2 (COX-2). Simultaneously, activation of the MAPK family—including extracellular signal-regulated kinase (ERK), p38 MAPK, and c-Jun N-terminal kinase (JNK)—further amplifies inflammatory signalling by regulating transcription factors such as AP-1 and increasing cytokine production. These interconnected pathways establish positive feedback mechanisms that sustain inflammation and contribute to chronic tissue injury [43,44].

**Figure 3:**
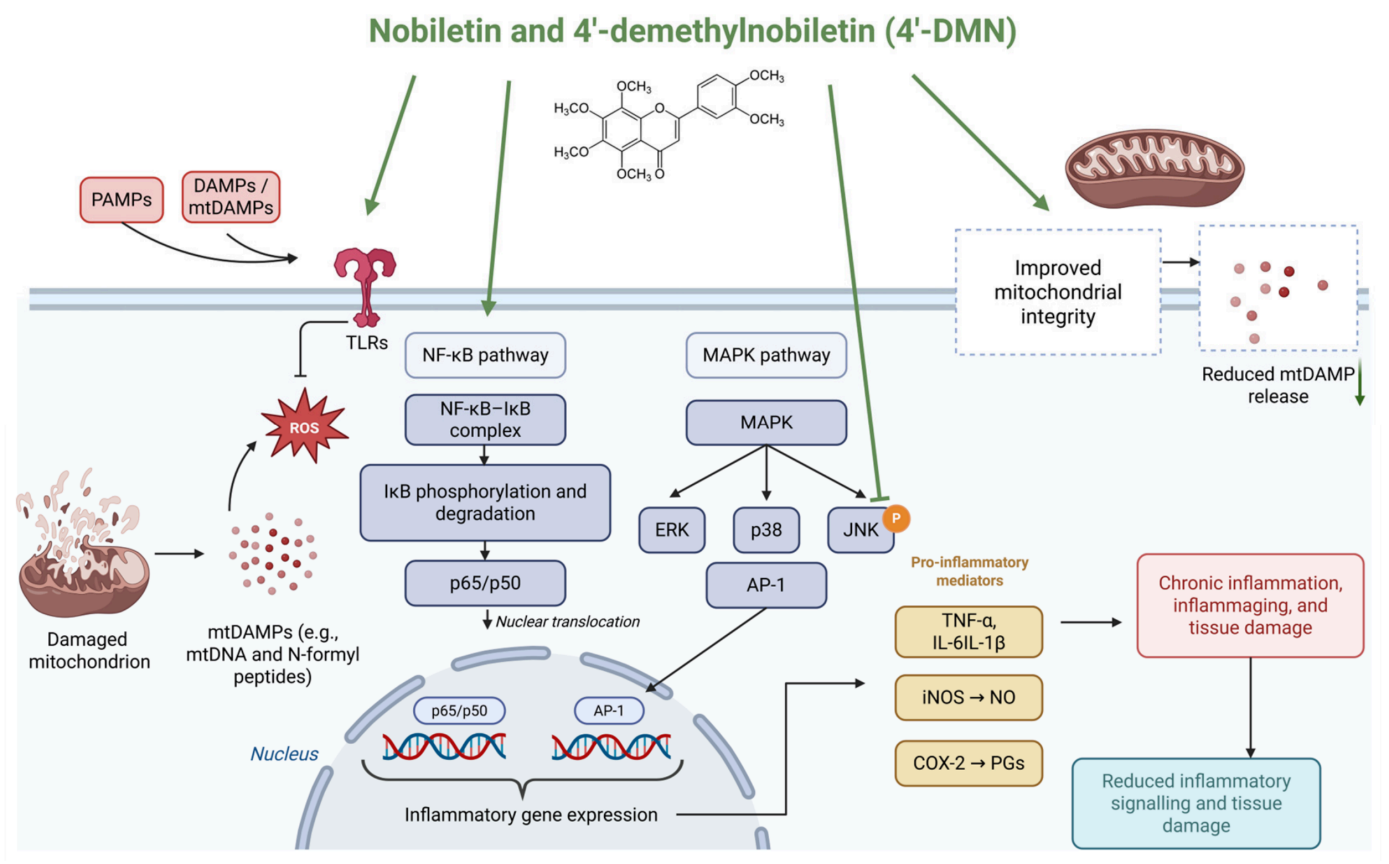
Anti-inflammatory mechanisms of nobiletin and 4′-demethylnobiletin. Abbreviations: AP-1, activator protein 1; COX-2, cyclooxygenase-2; DAMPs, damage-associated molecular patterns; ERK, extracellular signal-regulated kinase; IκB, inhibitor of NF-κB; IL, interleukin; iNOS, inducible nitric oxide synthase; JNK, c-Jun N-terminal kinase; MAPK, mitogen-activated protein kinase; mtDAMPs, mitochondrial DAMPs; mtDNA, mitochondrial DNA; NF-κB, nuclear factor kappa B; NO, nitric oxide; PAMPs, pathogen-associated molecular patterns; PGs, prostaglandins; ROS, reactive oxygen species; TLRs, Toll-like receptors; TNF-α, tumour necrosis factor alpha; 4′-DMN, 4′-demethylnobiletin.

Accumulating evidence indicates that nobiletin and its major metabolite, 4′-demethylnobiletin (4′-DMN), exert potent anti-inflammatory effects by simultaneously targeting multiple components of these signalling networks. Experimental studies have demonstrated that 4′-DMN inhibits activation of the NF-κB pathway by preventing the nuclear translocation of the p65 and p50 subunits, thereby suppressing transcription of genes encoding pro-inflammatory cytokines and inflammatory enzymes [45]. As a consequence, production of TNF-α, IL-1β, IL-6, and other inflammatory mediators is significantly reduced, attenuating both local and systemic inflammatory responses. In addition to inhibiting NF-κB activation, nobiletin suppresses inflammatory amplification by modulating the MAPK signalling pathway. In particular, inhibition of JNK phosphorylation reduces activation of downstream transcription factors responsible for inflammatory gene expression. This mechanism markedly reduces inducible iNOS expression, thereby lowering NO production. Although physiologically involved in vascular regulation and host defence, NO becomes cytotoxic when produced in excess during chronic inflammation. Similarly, nobiletin reduces COX-2 expression, thereby limiting prostaglandin synthesis and diminishing inflammatory pain, vascular permeability, and leukocyte recruitment [2]. The main inflammatory pathways targeted by nobiletin and its metabolite 4′-demethylnobiletin, including NF-κB and JNK–MAPK signalling, are summarised in Figure 3.

Beyond directly inhibiting inflammatory signalling pathways, nobiletin indirectly attenuates inflammation through its antioxidant properties. Excessive ROS generation enhances NF-κB activation and perpetuates inflammatory responses. By reducing intracellular ROS levels and improving mitochondrial function, nobiletin interrupts this reciprocal interaction between oxidative stress and inflammation. Preservation of mitochondrial integrity further limits the release of mitochondrial damage-associated molecular patterns (mtDAMPs), which are potent activators of innate immune responses and chronic sterile inflammation. Consequently, the antioxidant and anti-inflammatory activities of nobiletin act synergistically to restore cellular homeostasis [46,47].

The anti-inflammatory properties of nobiletin may be particularly relevant in the context of ageing, where persistent activation of inflammatory pathways contributes to endothelial dysfunction, insulin resistance, neurodegeneration, and tissue fibrosis. By suppressing the production of inflammatory cytokines and inhibiting key signalling pathways such as NF-κB and JNK-MAPK, nobiletin effectively disrupts the positive feedback loops that sustain chronic inflammation. Experimental studies consistently demonstrate dose-dependent reductions in inflammatory mediators following nobiletin administration, supporting its potential as a geroprotective compound that mitigates inflammaging and reduces the risk of age-related diseases [48,49,50].

### 3.3. Nobiletin and Cellular Senescence

Cellular senescence is a fundamental hallmark of ageing characterised by irreversible cell-cycle arrest, altered cellular metabolism, mitochondrial dysfunction, resistance to apoptosis, and the development of the senescence-associated secretory phenotype (SASP) [51]. The accumulation of senescent cells contributes to chronic low-grade inflammation, tissue dysfunction, impaired regeneration, and progression of numerous age-related diseases, including pulmonary fibrosis, neurodegenerative disorders, metabolic syndrome, and cardiovascular disease [52]. Consequently, pharmacological interventions that prevent the induction of senescence or attenuate the detrimental consequences of senescent cell accumulation have attracted substantial interest in geroscience.

Emerging evidence suggests that nobiletin may exert anti-senescent effects through multiple complementary mechanisms. One of the most direct demonstrations was reported by Li et al., who showed that nobiletin inhibited cellular senescence in A549 lung epithelial cells and attenuated fibrotic responses associated with idiopathic pulmonary fibrosis. Mechanistically, these effects were linked to modulation of the PI3K/AKT/MDM2/p53 signalling pathway, which plays a central role in regulating cellular senescence, DNA damage responses, and cell-cycle arrest. The authors also demonstrated reduced fibroblast migration, suggesting that nobiletin may counteract senescence-driven fibrosis-related tissue remodelling [53].

Additional support for nobiletin’s anti-senescent activity comes from experimental ageing models. In *Caenorhabditis elegans*, nobiletin significantly extended lifespan and enhanced resistance to oxidative and thermal stress. These beneficial effects were accompanied by improved cellular stress tolerance and attenuation of age-associated functional decline, suggesting that nobiletin may modulate fundamental pathways involved in biological ageing [16]. Although these observations were obtained in a non-mammalian model, they provide evidence that nobiletin influences conserved ageing-related mechanisms beyond simple antioxidant activity.

Oxidative stress is a major trigger of cellular senescence through activation of DNA damage responses, mitochondrial dysfunction, and pro-inflammatory signalling [54]. Numerous studies discussed in this review indicate that nobiletin reduces intracellular ROS generation and suppresses NF-κB-dependent inflammatory pathways, thereby potentially limiting the initiation and propagation of senescence-associated phenotypes [16,45]. By reducing oxidative and inflammatory stress, nobiletin may indirectly attenuate SASP formation, a principal driver of inflammaging and tissue deterioration during ageing.

**Figure 4:**
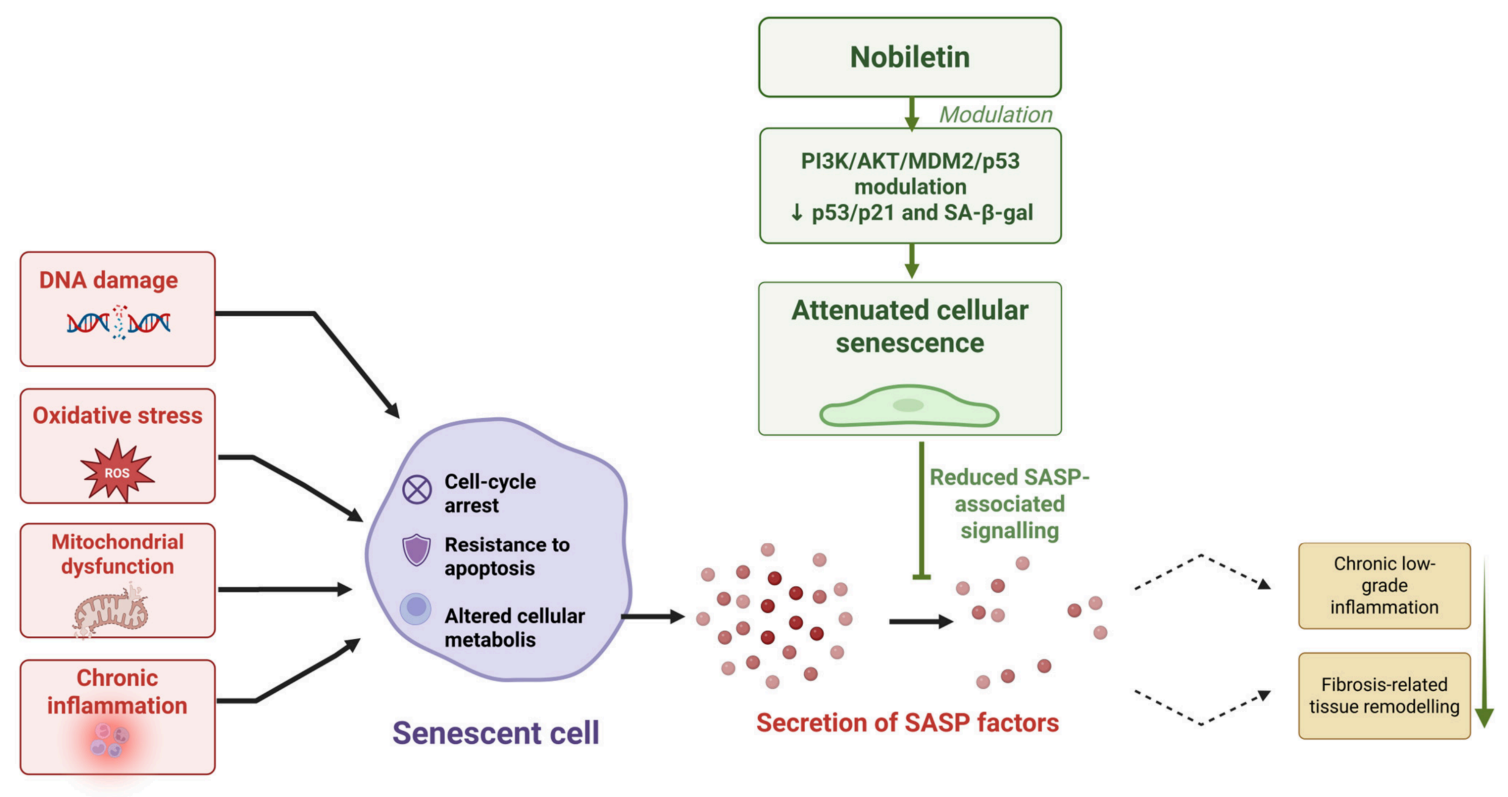
Proposed senomorphic effects of nobiletin. Abbreviations: AKT, protein kinase B; MDM2, mouse double minute 2 homolog; PI3K, phosphoinositide 3-kinase; SA-β-gal, senescence-associated β-galactosidase; SASP, senescence-associated secretory phenotype.

Another mechanism linking nobiletin to the prevention of senescence involves maintaining mitochondrial homeostasis. Mitochondrial dysfunction is increasingly recognised as a major contributor to the induction of senescence through excessive ROS production, metabolic dysregulation, and impaired energy metabolism. Nobiletin has been shown to enhance mitochondrial function by activating SIRT1/PGC-1α signalling and improving mitochondrial bioenergetics [55]. Furthermore, studies summarised in the present review indicate that nobiletin promotes mitophagy by activating the PINK1–Parkin pathway, facilitating the removal of dysfunctional mitochondria and reducing the accumulation of senescence-promoting cellular damage. The proposed mechanisms underlying the senomorphic effects of nobiletin, including its influence on cellular senescence and SASP-associated consequences, are presented in Figure 4.

Collectively, the currently available evidence suggests that nobiletin may act as a senomorphic rather than a classical senolytic compound. While direct elimination of senescent cells has not yet been demonstrated, nobiletin appears capable of reducing several key drivers of cellular senescence, including oxidative stress, chronic inflammation, mitochondrial dysfunction, and dysregulated stress responses. Nevertheless, direct investigations evaluating senescence biomarkers such as p16/INK4a, p21/CIP1/WAF1, SA-β-gal activity, and SASP mediators remain limited. Future studies should therefore clarify whether nobiletin can directly modulate senescent cell burden in vivo and determine its potential role within emerging gerotherapeutic strategies (Table 1).

### 3.4. Role of Nobiletin in Modulation of Circadian Rhythm and Metabolism

**Figure 5:**
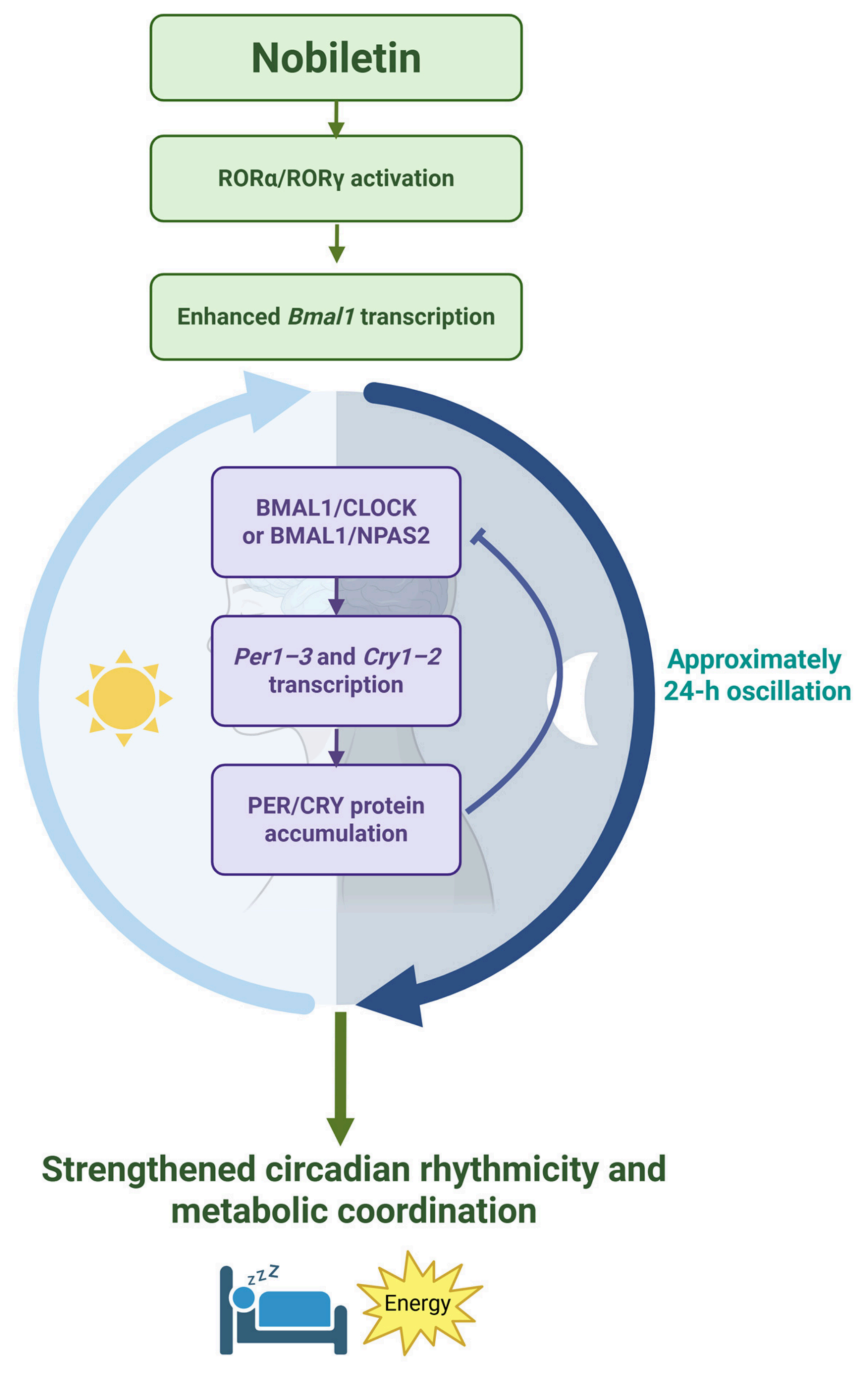
Proposed effect of nobiletin on circadian rhythm regulation. Abbreviations: BMAL1, brain and muscle ARNT-like protein 1; CLOCK, circadian locomotor output cycles kaput; CRY, cryptochrome; NPAS2, neuronal PAS domain protein 2; PER, period; RORα/RORγ, retinoic acid receptor-related orphan receptors α and γ.

The circadian system is a highly conserved endogenous timekeeping mechanism that synchronises physiological and metabolic processes with the 24-h light–dark cycle [57]. Virtually every mammalian cell contains an intrinsic molecular clock that regulates numerous biological functions, including sleep–wake cycles, hormone secretion, body temperature, energy metabolism, immune responses, and cellular repair processes [58]. Disruption of circadian rhythms has emerged as an important contributor to ageing and is associated with an increased risk of neurodegenerative diseases, obesity, insulin resistance, cardiovascular disorders, and metabolic syndrome [59]. Consequently, restoration of circadian homeostasis has become an attractive therapeutic target for promoting healthy ageing and extending healthspan. Recent evidence indicates that nobiletin functions as a natural modulator of the circadian clock by interacting with key components of the molecular oscillator. Experimental studies performed in animal models of Alzheimer’s disease (AD) have demonstrated that nobiletin effectively counteracts circadian disruption by restoring both behavioural and metabolic rhythmicity [60]. Administration of nobiletin significantly improved sleep architecture by reducing fragmented sleep episodes and increasing sleep consolidation, thereby restoring more regular sleep–wake cycles. Since sleep disturbances are among the earliest clinical manifestations of AD and are closely associated with accelerated cognitive decline, these findings suggest that circadian regulation may represent an important mechanism underlying the neuroprotective effects of nobiletin [20,61]. In addition to improving sleep behaviour, nobiletin has been shown to normalise several physiological parameters that exhibit circadian oscillations. In AD models, treatment restored normal patterns of oxygen consumption (VO_2_) and carbon dioxide production (VCO_2_), reflecting improved mitochondrial oxidative metabolism and correction of metabolic dysregulation. Furthermore, nobiletin stabilised daily fluctuations in core body temperature, one of the most robust physiological outputs of the circadian clock. Restoration of these rhythmic parameters indicates improved synchronisation between central and peripheral biological clocks, ultimately contributing to enhanced metabolic efficiency and maintenance of physiological homeostasis [62].At the molecular level, the effects of nobiletin are mediated through modulation of the core circadian transcription–translation feedback loop. The mammalian molecular clock consists of transcription factors such as BMAL1 (Brain and Muscle ARNT-Like 1) and CLOCK, which activate the expression of circadian target genes, including Period (Per1–3) and Cryptochrome (Cry1–2) [63]. These proteins subsequently inhibit BMAL1/CLOCK activity, thereby generating self-sustaining oscillations with an approximately 24-h periodicity. Nobiletin has been shown to enhance the expression levels of several core clock genes, including Bmal1, Npas2, and Rora, particularly in the cerebral cortex [62]. Increased expression and rhythmicity of these genes strengthen circadian oscillations and improve downstream regulation of numerous metabolic pathways involved in glucose utilisation, lipid metabolism, mitochondrial respiration, and cellular energy balance. An important molecular target of nobiletin appears to be the retinoic acid receptor-related orphan receptors (RORα and RORγ), which function as positive regulators of BMAL1 transcription. By acting as an agonist of these nuclear receptors, nobiletin enhances circadian gene expression and reinforces molecular clock function. Experimental studies have demonstrated that the beneficial metabolic effects of nobiletin are largely abolished in animals lacking an intact circadian clock, indicating that proper clock function is essential for mediating its therapeutic activity. The influence of nobiletin on circadian regulation has important metabolic consequences. Circadian clocks tightly regulate glucose homeostasis, insulin sensitivity, lipid oxidation, and energy expenditure. Circadian disruption promotes obesity, hepatic steatosis, insulin resistance, and type 2 diabetes mellitus by impairing the temporal coordination of metabolic pathways. In experimental models of metabolic syndrome, nobiletin improved glucose tolerance, enhanced insulin sensitivity, reduced adiposity, and protected against diet-induced obesity. These beneficial effects were accompanied by increased mitochondrial respiration, improved energy expenditure, and restoration of metabolic flexibility, suggesting that circadian regulation represents a central mechanism through which nobiletin improves systemic metabolism [64,65]. The proposed mechanism by which nobiletin strengthens the molecular circadian clock through RORα/RORγ and the BMAL1/CLOCK–PER/CRY feedback loop is illustrated in Figure 5.

Beyond its metabolic actions, restoration of circadian homeostasis may contribute to several additional geroprotective effects of nobiletin. Circadian regulation influences DNA repair, mitochondrial turnover, autophagy, antioxidant defence, immune function, and inflammatory responses. Therefore, enhancing molecular clock function may indirectly reduce oxidative stress, attenuate chronic inflammation, improve mitochondrial quality control, and preserve neuronal function during ageing. These pleiotropic actions support the notion that circadian modulation is a fundamental mechanism by which nobiletin promotes healthy ageing and protects against age-related diseases.

A summary of key preclinical studies relevant to the potential geroprotective effects of nobiletin is presented in Table 2.

## 4. Potential Therapeutic Applications

Extensive preclinical evidence indicates that nobiletin exerts antioxidant, anti-inflammatory, anti-apoptotic, metabolic, and cytoprotective effects, making it a promising candidate for the prevention and treatment of cardiovascular, metabolic, renal, hepatic, neurodegenerative, and neoplastic disorders [70]. Although most available evidence comes from experimental animal models and in vitro studies, the results consistently demonstrate that nobiletin targets several fundamental mechanisms of ageing, including oxidative stress, mitochondrial dysfunction, chronic inflammation, ferroptosis, and impaired cellular metabolism. Among the diseases in which nobiletin has demonstrated particularly promising therapeutic potential is type 2 diabetes mellitus, a metabolic disorder whose global prevalence continues to increase due to sedentary lifestyles and dietary changes [71]. Cardiovascular disease remains the leading cause of mortality among patients with diabetes, largely because chronic hyperglycemia promotes oxidative stress, endothelial dysfunction, mitochondrial impairment, abnormal autophagy, chronic inflammation, and disturbances in cellular redox homeostasis [72]. In recent years, ferroptosis has emerged as an additional mechanism contributing to diabetic cardiovascular complications. Ferroptosis is a regulated form of cell death characterised by excessive iron-dependent lipid peroxidation resulting from glutathione depletion and uncontrolled accumulation of ROS. These processes contribute to myocardial injury, impaired cardiac function, and accelerated progression of diabetic cardiomyopathy. Experimental studies have demonstrated that nobiletin effectively attenuates these pathological changes [73]. In a murine model of type 2 diabetes induced by a high-fat diet, treatment with nobiletin significantly improved cardiac function and reduced serum levels of creatine kinase (CK) and lactate dehydrogenase (LDH), both of which are established biomarkers of myocardial infarction [74].

Furthermore, nobiletin administration decreased malondialdehyde (MDA) concentrations, indicating reduced lipid peroxidation, while simultaneously lowering the myocardial apoptotic index [75]. These findings suggest that inhibition of oxidative stress and ferroptosis is a principal mechanism underlying nobiletin’s cardioprotective effects, supporting its potential to reduce cardiovascular complications associated with diabetes mellitus. Protective effects of nobiletin have also been demonstrated in chronic kidney disease (CKD), another disorder closely associated with ageing and metabolic dysfunction. CKD is characterised by progressive loss of renal function resulting from persistent inflammation, oxidative stress, fibrosis, and apoptosis, most commonly secondary to diabetes mellitus, hypertension, or chronic glomerulonephritis [76]. Sustained activation of transforming growth factor-beta (TGF-β) promotes extracellular matrix deposition and renal fibrosis, whereas increasing evidence implicates ferroptosis in the transition from acute kidney injury to chronic irreversible renal damage. Experimental investigations have shown that nobiletin significantly alleviates oxidative stress-induced renal injury by suppressing ferroptosis, reducing inflammatory responses, and attenuating apoptotic signalling pathways [77]. These effects preserve renal tissue architecture and slow disease progression. Although current findings primarily support a preventive rather than a curative role, they suggest that modulating oxidative stress and ferroptosis may be an effective strategy for limiting chronic kidney injury. However, further studies are required to determine whether nobiletin can reverse established renal fibrosis or improve renal function in advanced disease stages.

Similarly, nobiletin has shown considerable therapeutic potential in liver diseases, particularly alcohol-related liver disease (ALD). Chronic alcohol consumption induces excessive production of acetaldehyde and ROS during hepatic metabolism, resulting in mitochondrial dysfunction, lipid peroxidation, chronic inflammation, steatosis, fibrosis, and ultimately hepatocellular carcinoma. Because mitochondria represent a major target of alcohol-induced toxicity, preservation of mitochondrial integrity is essential for preventing disease progression. Experimental studies have demonstrated that nobiletin supplementation effectively reduces hepatic oxidative stress and inflammatory responses while simultaneously improving mitochondrial function by activating the hepatic NRF1-TFAM signalling pathway, which regulates mitochondrial biogenesis and mitochondrial DNA maintenance [78]. Consequently, nobiletin attenuates liver injury, reduces fibrosis, and preserves hepatocellular viability, highlighting its potential as a hepatoprotective agent in chronic liver diseases. Atherosclerosis represents another chronic inflammatory disorder in which nobiletin exhibits beneficial biological activity. The development of atherosclerotic plaques is initiated by endothelial dysfunction and oxidation of low-density lipoproteins (LDL), leading to macrophage recruitment, foam cell formation, and chronic vascular inflammation [79]. Hyperlipidemia, hypertension, obesity, smoking, and diabetes mellitus accelerate this process through increased oxidative stress and persistent activation of inflammatory pathways [80]. Nobiletin exerts anti-atherosclerotic effects through several complementary mechanisms. By improving insulin sensitivity and glucose metabolism, it indirectly reduces vascular injury associated with chronic hyperglycemia. Simultaneously, its antioxidant activity inhibits LDL oxidation, thereby decreasing the formation of pro-inflammatory oxidised LDL particles [81]. Furthermore, nobiletin suppresses macrophage lipid uptake by regulating the PPARγ/CD36 signalling pathway, thereby limiting foam cell formation and slowing the progression of atherosclerotic lesions. These findings support the potential use of nobiletin to prevent cardiovascular diseases associated with metabolic syndrome and ageing.

Beyond its cardiometabolic effects, nobiletin has emerged as a promising natural compound in cancer prevention and therapy. Chronic inflammation, oxidative stress, dysregulated apoptosis, and immune evasion constitute major hallmarks of carcinogenesis that flavonoids can modulate [82]. Among various malignancies, non-small cell lung cancer (NSCLC) has been extensively investigated in relation to nobiletin treatment. NSCLC frequently overexpresses programmed death-ligand 1 (PD-L1), an immune checkpoint protein that suppresses cytotoxic T-cell activity and enables tumour cells to evade immune surveillance. Experimental studies have demonstrated that nobiletin significantly downregulates PD-L1 expression by modulating the miR-197/STAT3/PD-L1 signalling pathway, thereby enhancing antitumor immune responses. In addition, nobiletin inhibits epithelial-mesenchymal transition (EMT), suppresses tumour cell migration and invasion, induces apoptosis, and inhibits malignant cell proliferation. It has also demonstrated chemopreventive activity by reducing 4-(methylnitrosamino)-1-(3-pyridyl)-1-butanone (NNK)-induced lung tumorigenesis, suggesting potential utility in preventing tobacco-related cancers. These pleiotropic anticancer effects indicate that nobiletin may not only function as a direct antineoplastic agent but also enhance the efficacy of conventional chemotherapy while potentially reducing treatment-associated toxicity [83,84,85].

Collectively, the available evidence indicates that nobiletin exerts broad therapeutic activity by coordinating the regulation of oxidative stress, mitochondrial homeostasis, inflammatory signalling, ferroptosis, apoptosis, and cellular metabolism. These mechanisms underlie its protective effects across multiple organ systems and support its classification as a promising geroprotective phytochemical with potential applications in preventing age-related chronic diseases. Nevertheless, despite compelling results obtained in experimental models, translation into clinical practice remains limited. Most studies have been conducted in vitro or in animal models, and robust clinical trials evaluating the efficacy, pharmacokinetics, optimal dosage, long-term safety, and therapeutic effectiveness of nobiletin in humans are still lacking. Future clinical research will therefore be essential to determine whether the encouraging preclinical findings can be successfully translated into effective therapeutic strategies to extend healthspan and reduce the burden of ageing-associated diseases.

## 5. Human Studies and Clinical Evidence

Despite extensive preclinical research supporting the anti-ageing and disease-modifying potential of nobiletin, evidence from human studies remains surprisingly limited. Most available investigations have focused on pharmacokinetics, metabolism, safety assessment, or citrus-derived flavonoid mixtures enriched in polymethoxyflavones, a group of highly methoxylated flavones that includes nobiletin, tangeretin, sinensetin, and heptamethoxyflavone, rather than on purified nobiletin administered as a single therapeutic agent. Consequently, direct clinical evidence supporting the use of nobiletin for healthy ageing interventions remains insufficient.

Human metabolic studies have demonstrated that nobiletin undergoes extensive hepatic biotransformation following oral administration. In particular, demethylation by cytochrome P450 enzymes generates several biologically active metabolites, including 3′- and 4′-demethylnobiletin, which may substantially contribute to the biological activity observed in vivo [86]. Furthermore, existing pharmacokinetic studies suggest that the systemic availability of the parent compound remains relatively low because of poor aqueous solubility and extensive first-pass metabolism [87,88].

Indirect clinical evidence has emerged from studies evaluating citrus flavonoid-rich dietary patterns and polymethoxyflavone-containing preparations. Epidemiological investigations have reported associations between higher flavonoid intake and improved cardiometabolic health, reduced arterial stiffness, and a lower prevalence of hypertension [8,9]. Nevertheless, these studies evaluated total flavonoid exposure rather than nobiletin specifically, thereby preventing causal attribution to a single compound.

Several reviews have highlighted the substantial translational gap between encouraging preclinical findings and the currently available human evidence. Although nobiletin exhibits promising neuroprotective, anti-inflammatory, metabolic, and cardioprotective effects in experimental models, well-designed randomised controlled trials evaluating its efficacy in ageing-related conditions remain largely absent [4,17,38].

Therefore, future clinical studies should focus on determining optimal dosing regimens, formulation-dependent bioavailability, long-term safety, pharmacokinetic variability, and clinically meaningful biomarkers of biological ageing. Such investigations are essential before nobiletin can be considered a validated geroprotective intervention in humans (Table 3).

Although preclinical studies provide substantial evidence supporting the geroprotective potential of nobiletin, clinical evidence remains limited. At least one registered clinical trial has evaluated a citrus polymethoxyflavonoid-enriched intervention containing nobiletin together with other flavonoids, such as sinensetin and tangeretin; however, this study does not assess isolated nobiletin as a single active compound. Therefore, well-designed clinical trials using standardised nobiletin formulations are still required to determine its efficacy, safety, pharmacokinetics, optimal dosage, and therapeutic relevance in humans.

## 6. Bioavailability

### 6.1. Physicochemical and Pharmacokinetic Limitations

Despite its remarkable pharmacological potential, the clinical application of nobiletin remains considerably limited by its poor oral bioavailability. Oral administration represents the most convenient and preferred route for long-term therapeutic use; however, several physicochemical and biological factors restrict the efficient absorption and systemic distribution of this polymethoxylated flavone [70]. Although nobiletin possesses relatively high membrane permeability owing to its lipophilic nature, its poor aqueous solubility, extensive first-pass metabolism, and variable intestinal biotransformation collectively reduce the amount of the biologically active compound that reaches the systemic circulation. Consequently, improving the bioavailability of nobiletin has become a major challenge in its pharmaceutical development. The limited intestinal absorption of nobiletin has been demonstrated in experimental studies using the rat single-pass intestinal perfusion model. In these experiments, fasted and anesthetised animals underwent isolated jejunal or ileal perfusion with Krebs-Ringer solution containing nobiletin while maintaining normal intestinal blood flow [87]. Analysis of intestinal perfusates together with portal vein plasma samples revealed relatively poor absorption of nobiletin across the intestinal epithelium. These findings indicate that dissolution in the gastrointestinal tract, rather than membrane permeability, is the principal rate-limiting step in systemic absorption. Similar limitations are expected in humans, where oral administration results in relatively low plasma concentrations and limited therapeutic exposure despite favourable biological activity observed in experimental models.

From a physicochemical perspective, the bioavailability of nobiletin is largely determined by its molecular structure. As a highly methoxylated flavone, nobiletin exhibits pronounced lipophilicity and very low water solubility. Although the six methoxy substituents substantially increase membrane permeability and facilitate passive diffusion across biological membranes, including the blood–brain barrier, they simultaneously reduce dissolution in the aqueous environment of the gastrointestinal tract. Consequently, only a limited fraction of orally administered nobiletin becomes available for intestinal absorption before being eliminated from the digestive tract. Nevertheless, the high degree of methylation also confers greater chemical stability and oxidation resistance than in hydroxylated flavonoids, allowing nobiletin to remain biologically active for longer after absorption. Beyond limited dissolution, the metabolic fate of nobiletin substantially influences its systemic bioavailability [88]. Following intestinal absorption, nobiletin undergoes extensive first-pass metabolism in both the intestinal mucosa and the liver. Cytochrome P450 enzymes, particularly members of the CYP1A and CYP3A families, catalyse sequential O-demethylation reactions that generate several biologically active metabolites, including 3′-demethylnobiletin (3′-DMN), 4′-demethylnobiletin (4′-DMN), 3′,4′-demethylnobiletin (3′,4′-DMN), and 5′-demethylnobiletin (5′-DMN). Unlike many xenobiotics whose metabolites exhibit reduced biological activity, these metabolites retain—and in some experimental models even exceed—the pharmacological efficacy of the parent compound. Several studies have demonstrated that demethylated metabolites exhibit potent antioxidant, anti-inflammatory, neuroprotective, and anticancer activities, suggesting that the therapeutic effects of nobiletin arise from the combined activity of the parent molecule and its metabolites. Subsequent phase II metabolism involves glucuronidation and sulfation, which facilitate renal and biliary elimination but simultaneously decrease circulating concentrations of free nobiletin available to target tissues [86].

### 6.2. Role of the Gut Microbiota and Intestinal Transport

An additional factor increasingly recognised as a determinant of nobiletin bioavailability is the gut microbiota. The intestinal microbiome actively participates in the biotransformation of dietary polyphenols through enzymatic reactions, including demethylation, dehydroxylation, hydrolysis, and ring cleavage. These microbial transformations generate secondary metabolites that often differ substantially in their absorption, tissue distribution, and biological activity compared with the parent compound. Although the precise microbial metabolism of nobiletin has not yet been fully characterised, accumulating evidence suggests that interindividual differences in gut microbiota composition may contribute to considerable variability in its pharmacokinetics and therapeutic efficacy. Moreover, reciprocal interactions appear to exist, as flavonoids, including nobiletin, may beneficially modulate gut microbial composition by promoting the growth of beneficial bacterial genera while suppressing potentially pathogenic microorganisms. Such bidirectional interactions between nobiletin and the intestinal microbiome may influence systemic inflammation, intestinal barrier integrity, metabolic homeostasis, and immune function, thereby contributing indirectly to its geroprotective effects [92,93].

Another important determinant of oral bioavailability is intestinal transport. In addition to passive diffusion, flavonoids may interact with membrane transport proteins, including ATP-binding cassette (ABC) transporters such as P-glycoprotein (P-gp) and breast cancer resistance protein (BCRP). These efflux transporters can actively transport absorbed molecules back into the intestinal lumen, thereby limiting net absorption. Although the precise interaction of nobiletin with these transporters remains under investigation, modulation of transporter activity has been proposed as another mechanism influencing its pharmacokinetic profile and tissue distribution. Because poor solubility is a principal limitation to nobiletin absorption, numerous formulation strategies have been developed to improve its bioavailability. Among the most promising approaches are lipid-based delivery systems, nanoemulsions, phospholipid complexes, polymeric nanoparticles, liposomes, and self-microemulsifying drug delivery systems (SMEDDS). These technologies increase the apparent solubility of nobiletin, enhance intestinal dissolution, and facilitate transport across the intestinal epithelium [94,95].

### 6.3. Formulation and Delivery Strategies

Particularly encouraging results have been obtained using nanoemulsions formulated with Antarctic krill oil (AKO) enriched in docosahexaenoic acid (DHA)-containing phosphatidylcholine. The phospholipids present in AKO possess excellent amphiphilic properties, enabling spontaneous formation of micellar structures that effectively encapsulate nobiletin. These nanoemulsions enhance lipid digestion and lipolysis, promoting more efficient release of nobiletin during gastrointestinal digestion and facilitating rapid absorption into the bloodstream. Experimental studies demonstrated significantly improved tissue distribution and systemic bioavailability within two hours following oral administration compared with conventional formulations [96]. Similarly, SMEDDS have demonstrated substantial improvements in intestinal permeability and nobiletin absorption in animal models. These formulations spontaneously generate fine oil-in-water emulsions upon contact with gastrointestinal fluids, thereby increasing the surface area available for absorption and improving dissolution kinetics. Experimental studies in rats confirmed markedly enhanced intestinal uptake compared with crystalline nobiletin, suggesting that SMEDDS represent a promising platform for future oral formulations [97].

Nanotechnology has further expanded the possibilities for improving nobiletin delivery. Encapsulation within biodegradable nanoparticles, such as zein, sunflower pollen-derived carriers, or metal-polyphenol complexes, has been shown to improve chemical stability, protect nobiletin from premature metabolism, prolong controlled release, and enhance systemic exposure. These nanocarriers also improve pharmacokinetic profiles by increasing circulation time and facilitating tissue-specific delivery. Alternative approaches, including ionic liquid formulations, have also demonstrated markedly improved transdermal delivery, bypassing gastrointestinal degradation and first-pass hepatic metabolism while achieving substantially greater bioavailability than conventional crystalline preparations [98].

The development of advanced delivery systems should remain a priority. Nanocarriers, phospholipid-based formulations, lipid nanoparticles, self-microemulsifying delivery systems, and other precision delivery platforms have shown promising results in enhancing the absorption and tissue distribution of nobiletin. Continued optimisation of these technologies may significantly enhance therapeutic efficacy and facilitate future clinical application [96,97,98].

## 7. Challenges and Future Applications

### 7.1. Extraction, Standardisation, and Sustainable Production

Nobiletin is predominantly obtained from citrus peels, especially from species rich in polymethoxylated flavones, including mandarins, tangerines, and related citrus varieties. Citrus peel represents an abundant agro-industrial by-product generated during juice and food processing, making it an attractive and sustainable raw material for the recovery of bioactive compounds. However, extracting nobiletin is technically challenging because citrus peels contain a complex matrix of essential oils, organic acids, pectins, pigments, fibres, and other flavonoids, including tangeretin, sinensetin, hesperidin, and narirutin. The coexistence of structurally similar compounds necessitates carefully optimised extraction and purification procedures to obtain nobiletin of adequate purity for biological, nutraceutical, or pharmaceutical applications.

Conventional extraction of nobiletin is typically performed with organic solvents such as methanol, ethanol, or acetone, or their aqueous mixtures. The efficiency of extraction depends on several parameters, including solvent polarity, solid-to-liquid ratio, extraction time, temperature, particle size, and pH. Ethanol-based extraction is particularly attractive for food and nutraceutical applications because of its lower toxicity and greater regulatory acceptability compared with methanol or chlorinated solvents. Nevertheless, conventional maceration and reflux extraction may require long extraction times, high solvent volumes, and elevated temperatures, which can increase production costs and may promote degradation of thermolabile compounds.

Following extraction, purification is generally required to separate nobiletin from other citrus constituents. Chromatographic techniques, including silica gel column chromatography, preparative high-performance liquid chromatography, and adsorption resin-based fractionation, are commonly used to obtain purified polymethoxylated flavones. Although these approaches are effective, they may be time-consuming, costly, and difficult to scale up. Therefore, future industrial applications require the development of more efficient, selective, and environmentally sustainable purification strategies.

In recent years, green extraction technologies have gained increasing attention as alternatives to conventional solvent-based methods. Ultrasound-assisted extraction can improve mass transfer and reduce extraction time by disrupting plant cell walls, whereas microwave-assisted extraction can enhance solvent penetration and accelerate the release of intracellular compounds. Pressurised liquid extraction and supercritical fluid extraction, particularly with supercritical carbon dioxide and suitable co-solvents, may further reduce organic solvent consumption and improve process sustainability. These methods are particularly relevant to the valorisation of citrus-processing waste and may facilitate the scalable production of nobiletin-rich extracts [99,100].

### 7.2. Clinical Translation and Trial Design

Another challenge is the limited availability of human clinical data. Most mechanistic evidence has been obtained from in vitro experiments or animal models, whereas randomised controlled clinical trials remain scarce. Consequently, the optimal therapeutic dose, treatment duration, long-term safety, pharmacokinetic variability, and efficacy across different patient populations remain largely unknown. Standardisation of clinical study protocols and the identification of reliable pharmacodynamic biomarkers will be essential to translate encouraging preclinical findings into clinical practice. Safety considerations should also be addressed before widespread clinical application. Although preclinical toxicological studies suggest a relatively favourable safety profile, toxic effects have been observed in mice at doses exceeding approximately 540 mg/kg body weight [101]. Existing human studies largely focus on metabolism, pharmacokinetics, or dietary flavonoid exposure, and no completed trials have conclusively demonstrated beneficial effects on healthspan, frailty, cognitive ageing, or other clinically relevant ageing-related outcomes. Consequently, the relationship between the promising biological effects observed in experimental models and meaningful improvements in human ageing remains unclear [86,87,88].

### 7.3. Safety and Potential Drug Interactions

Excessive occupational exposure through inhalation or direct contact with the skin or eyes may result in local irritation. Long-term systemic toxicity, reproductive safety, and potential developmental effects have not yet been sufficiently investigated in humans; therefore, nobiletin supplementation cannot currently be recommended during pregnancy or lactation. Since hepatic metabolism and renal excretion represent the primary elimination pathways, careful monitoring of liver and kidney function may be advisable during prolonged supplementation, particularly in patients with pre-existing organ dysfunction.

An additional aspect requiring consideration is the potential for food–drug and herb–drug interactions. Nobiletin and its metabolites may alter the activity of cytochrome P450 enzymes and membrane transporters involved in drug disposition, potentially affecting the pharmacokinetics of statins, immunosuppressants, anticoagulants, antidepressants, and certain anticancer drugs. Although clinically significant interactions have not yet been conclusively demonstrated, further pharmacokinetic studies are warranted before recommending high-dose supplementation, especially in elderly patients receiving multiple medications. Future research should also explore precision delivery strategies, including ligand-targeted nanoparticles, mitochondria-targeting delivery systems, and stimuli-responsive nanocarriers that release nobiletin specifically within inflamed or senescent tissues. Combination therapies with other geroprotective compounds, such as resveratrol, fisetin, quercetin, metformin, or spermidine, may provide synergistic effects through complementary modulation of oxidative stress, autophagy, mitochondrial quality control, and cellular senescence. Moreover, advances in systems biology, metabolomics, transcriptomics, and artificial intelligence-assisted drug design may facilitate the identification of patient populations most likely to benefit from nobiletin therapy and accelerate the optimisation of novel formulations.

### 7.4. Ageing Biomarkers and Cellular Senescence

Another important limitation concerns the insufficient use of ageing-specific biomarkers in current research. Future studies should evaluate biological rather than chronological ageing by incorporating markers associated with cellular senescence, inflammaging, mitochondrial dysfunction, oxidative stress, and epigenetic ageing. Such an approach would provide a more precise assessment of nobiletin’s geroprotective potential and facilitate direct comparison with other emerging gerotherapeutic strategies. Moreover, none of the currently available human studies was specifically designed to assess recognised geroscience endpoints, which represents a major gap in the field [41,43]. Additional investigations are required to clarify the role of nobiletin in cellular senescence. Current evidence suggests that nobiletin primarily functions as a senomorphic agent by attenuating oxidative stress, chronic inflammation, mitochondrial dysfunction, and the development of the senescence-associated secretory phenotype, rather than directly eliminating senescent cells. However, studies evaluating established senescence biomarkers, including p16/INK4a, p21/CIP1/WAF1, SA-β-gal activity, and SASP-related mediators, remain limited. Determining whether nobiletin can directly influence senescent cell burden in vivo will be essential for defining its role among emerging gerotherapeutic interventions [16,55,98].

### 7.5. Pharmacokinetic and Microbiome-Related Variability

Further research is also needed to better understand the pharmacokinetic behaviour of nobiletin. Although poor aqueous solubility, extensive first-pass metabolism, and low systemic bioavailability are well recognised, the influence of age, sex, metabolic status, formulation type, and interindividual variability on therapeutic exposure remains insufficiently characterised. In addition, increasing evidence indicates that biologically active metabolites may substantially contribute to the observed therapeutic effects, highlighting the need for comprehensive pharmacokinetic-pharmacodynamic studies in humans [86,87].

The interaction between nobiletin and the gut microbiota is another promising but underexplored research area. Emerging evidence suggests that intestinal microorganisms participate in the biotransformation of nobiletin into metabolites with potentially distinct biological activities, while nobiletin itself may beneficially modulate microbial composition and function. A deeper understanding of these bidirectional interactions may help explain interindividual differences in treatment response and support the future development of personalised geroprotective interventions [92,93].

Overall, future research should focus on integrating rigorous clinical investigation with ageing biomarker assessment, advanced pharmacokinetic analyses, microbiome profiling, and next-generation delivery technologies. Such multidisciplinary approaches will be essential to determine whether the impressive benefits of nobiletin observed in experimental systems can be successfully translated into effective, evidence-based strategies to extend human healthspan and promote healthy longevity.

## 8. Conclusions

Population ageing represents one of the most pressing challenges facing modern healthcare systems, prompting an intensive search for interventions capable of extending not only lifespan but, more importantly, healthspan. Among naturally occurring phytochemicals, nobiletin has emerged as a particularly promising candidate owing to its broad spectrum of biological activities and its ability to modulate multiple interconnected pathways involved in the ageing process. Accumulating evidence indicates that nobiletin exerts antioxidant, anti-inflammatory, neuroprotective, cardiometabolic, and cytoprotective effects, while simultaneously influencing mitochondrial quality control, circadian rhythm regulation, autophagy, and cellular stress responses. These pleiotropic actions position nobiletin among the most promising flavonoid-derived compounds currently under investigation in the field of geroscience.

Importantly, the biological activity of nobiletin extends beyond a single molecular target. By attenuating oxidative stress and inflammaging, enhancing mitophagy through PINK1–Parkin signalling, improving mitochondrial function, regulating circadian oscillators, and potentially limiting cellular senescence, nobiletin simultaneously addresses several hallmarks of ageing. Such multitarget activity distinguishes it from conventional pharmacological approaches and supports the concept that nobiletin may contribute to the prevention or delay of age-related functional decline across multiple organ systems. Nevertheless, the strength of the evidence remains highly heterogeneous, with most data coming from in vitro and animal studies rather than human studies.

Despite robust preclinical support, the clinical translation of nobiletin remains in its early stages. Limited oral bioavailability, extensive first-pass metabolism, insufficient pharmacokinetic characterisation, and a lack of well-designed randomised clinical trials continue to represent major barriers to its therapeutic application. Furthermore, critical questions regarding optimal dosing, long-term safety, interindividual variability, interactions with the gut microbiota, and clinically relevant biomarkers of ageing remain largely unanswered. Consequently, current evidence supports classifying nobiletin as a promising geroprotector rather than a clinically validated gerotherapeutic intervention.

Future research should prioritise human intervention studies, advanced formulation strategies, and biomarker-driven clinical investigations to evaluate biological rather than chronological ageing. The integration of pharmacology, nanotechnology, systems biology, metabolomics, and artificial intelligence may further facilitate the development of personalised nobiletin-based interventions. Only through rigorous translational and clinical research will it be possible to determine whether the remarkable benefits observed in experimental models translate into meaningful improvements in human healthspan and resilience to age-associated diseases.

In summary, nobiletin is among the most promising citrus-derived flavonoids in the field of healthy ageing research. Its ability to simultaneously target mitochondrial dysfunction, chronic inflammation, circadian dysregulation, oxidative stress, and cellular senescence provides a compelling biological rationale for further investigation. However, definitive clinical validation remains necessary before nobiletin can be incorporated into evidence-based strategies for the prevention of ageing-related disorders and the promotion of healthy longevity.

## Figures and Tables

**Table 1:** Evidence supporting nobiletin’s anti-senescent activity.

Biological Effect	Experimental Model	Dose/Concentration and Treatment Duration	Comparator/Positive Control	Main Findings	Evidence Level	References
Inhibition of cellular senescence	A549 lung epithelial cells	20 μM nobiletin; senescence induced with bleomycin 50 μg/mL	Bleomycin/model control; positive control not reported	Reduced senescence and fibrosis-related responses via PI3K/AKT/MDM2/p53 signalling	In vitro	[53]
Lifespan extension	*Caenorhabditis elegans*	3.13, 6.25, and 12.5 μM nobiletin	Untreated/DMSO control; positive control not reported	Increased lifespan and stress resistance	In vivo (non-mammalian model)	[16]
Reduction of oxidative stress	*Caenorhabditis elegans*	3.13, 6.25, and 12.5 μM nobiletin	Untreated/DMSO control; positive control not reported	Decreased ROS accumulation and improved antioxidant defence	In vivo (non-mammalian model)	[16]
Anti-inflammatory activity	Multiple experimental models	model-dependent; reported in vitro concentrations include 3.75–60 μM nobiletin in RAW264.7 macrophages	LPS-stimulated/model control; positive control not reported	Suppression of NF-κB signalling and inflammatory mediators	in vitro and in vivo	[56]
Improvement of mitochondrial function	Porcine oocytes	5, 10, 25, and 50 μM nobiletin; 44 h culture	Untreated/control oocytes; positive control not reported	Activation of SIRT1/PGC-1α signalling and enhanced mitochondrial activity	In vitro	[55]

AKT, protein kinase B; DMSO, dimethyl sulfoxide; LPS, lipopolysaccharide; MDM2, mouse double minute 2 homolog; NF-κB, nuclear factor kappa B; p53, tumour protein p53; PGC-1α, peroxisome proliferator-activated receptor gamma coactivator 1-alpha; PI3K, phosphoinositide 3-kinase; RAW264.7, murine macrophage cell line; ROS, reactive oxygen species; SIRT1, sirtuin 1. The evidence level was classified according to the experimental model used in each cited study. Cell-based experiments were classified as in vitro; animal or whole-organism studies as in vivo; and studies combining more than one model type as in vitro and in vivo, depending on the model.

**Table 2:** Summary of experimental and observational studies investigating the anti-ageing effects of nobiletin.

Study/Model	Mechanism of Action	Dosage	Effects on Ageing	Key Findings	References
Mice with metabolic disorder	Activation of circadian rhythms via RORα/RORγ receptors	Nobiletin 0.1% in the diet for 8 weeks	Improved metabolism, weight reduction, and protection from metabolic syndrome	Nobiletin enhances circadian rhythms, thereby improving energy homeostasis and protecting against metabolic dysfunctions.	[19]
Aged mice on a high-fat diet	Enhancement of mitochondrial function in skeletal muscle	Nobiletin 0.1% in the diet for 12 weeks	Improved physical endurance, increased ATP production, and reduced oxidative stress.	Nobiletin increases the expression of genes involved in the electron transport chain, enhancing mitochondrial function and improving age-related metabolic health.	[21]
*Caenorhabditis elegans* model	Reduction of oxidative stress, mitochondrial function improvement	50 µM nobiletin in culture medium	Lifespan extension, increased stress resistance	Nobiletin delays ageing by reducing oxidative stress and enhancing mitochondrial function, thereby extending lifespan in nematodes.	[16]
Triple transgenic Alzheimer’s mouse model (3xTg-AD)	Reduction of soluble β-amyloid levels, improvement in cognitive function	Nobiletin 50 mg/kg, oral gavage daily for 4 weeks	Memory improvement, cognitive enhancement	Nobiletin lowers β-amyloid levels in the brain and improves memory and learning, suggesting its potential as a therapeutic agent for Alzheimer’s disease.	[66]
Porcine oocytes (in vitro)	Regulation of the SIRT1/PGC-1α signalling pathway, enhancement of mitochondrial function	50 µM nobiletin treatment for 24 h	Enhanced oocyte maturation, reduced oxidative stress	Nobiletin improves oocyte maturation by mitigating oxidative stress and boosting mitochondrial efficiency, which may be relevant in reproductive ageing and fertility.	[55]
Chicken granulosa cells (in vitro)	Activation of AMPK/SIRT1 pathways, promotion of autophagy and mitophagy	10 µM nobiletin for 24 h	Reduction of oxidative stress, improved cellular longevity	Nobiletin significantly reduced oxidative damage and increased antioxidant capacity, thereby promoting cellular longevity and reducing markers of ageing.	[15]
Chicken ovarian follicles (in vivo)	Enhancement of mitophagy via PINK1 and Parkin pathways, suppression of NF-κB signalling	Nobiletin 50 mg/kg oral daily for 30 days	Amelioration of ageing-associated ovarian decline	Nobiletin improved the structural integrity and functional health of ovarian follicles, suggesting a potential to enhance fertility lifespan.	[15]
C2C12 myoblast cells (in vitro)	Improvement of mitochondrial function, reduction of oxidative stress	10 µM nobiletin treatment for 48 h	Prevention of cellular ageing	Nobiletin prevented D-galactose-induced senescence by enhancing mitochondrial biogenesis and reducing ROS levels.	[67]
D-galactose-induced ageing in mice (skeletal muscle)	Regulation of protein homeostasis pathways	50 mg/kg nobiletin i.p. daily for 6 weeks	Improvement of muscle mass and function	Nobiletin alleviated skeletal muscle atrophy by enhancing proteostasis and mitochondrial health in ageing mice.	[68]
D-galactose-induced ageing mice (hippocampus)	Enhancement of hippocampal neurogenesis	50 mg/kg oral daily for 6 weeks	Amelioration of memory impairment	Nobiletin improved learning and memory abilities by promoting hippocampal neurogenesis and reducing oxidative stress.	[69]

3xTg-AD, triple-transgenic mouse model of Alzheimer’s disease; AD, Alzheimer’s disease; AMPK, AMP-activated protein kinase; ATP, adenosine triphosphate; i.p., intraperitoneal administration; NF-κB, nuclear factor kappa B; PGC-1α, peroxisome proliferator-activated receptor gamma coactivator 1-alpha; PINK1, PTEN-induced kinase 1; ROS, reactive oxygen species; RORα/RORγ, retinoic acid receptor-related orphan receptors alpha and gamma; SIRT1, sirtuin 1.

**Table 3:** Summary of epidemiological and clinical evidence relevant to the potential health effects of nobiletin.

Study Category	Population	Dose/Formulation and Treatment Duration	Main Findings	Limitations	Evidence Level	References
PERSIAN cohort study (flavonoid intake)	Adult population	Not applicable—observational assessment of dietary flavonoid intake; no nobiletin intervention.	Higher flavonoid intake is not associated with lower arterial stiffness and better vascular health indicators.	Nobiletin has not been evaluated individually	Epidemiological	[8]
US population study	Adult population	Not applicable—observational assessment of dietary flavonoid intake; no nobiletin intervention.	Dietary flavonoids are associated with a lower prevalence of hypertension	Observational design; no specific assessment of nobiletin	Epidemiological	[9]
Multicentre, randomised, double-blind, placebo-controlled trial	Healthy Japanese adults aged ≥ 65 years with subjective memory complaints; 108 participants included in the per-protocol analysis	Nobilex^®^ provides 30 mg nobiletin and 17.4 mg tangeretin daily, together with *Kaempferia parviflora* and *Peucedanum japonicum*; 16 weeks.	A multicomponent Nobilex^®^ supplement providing 30 mg nobiletin and 17.4 mg tangeretin daily for 16 weeks improved the total WMS-R score and selected memory domains compared with placebo.	Multicomponent formulation containing tangeretin and additional plant materials; per-protocol analysis; short intervention period; effects cannot be attributed specifically to nobiletin	Randomised clinical evidence	[89]
Pilot-controlled intervention study	Six patients with mild-to-moderate Alzheimer’s disease receiving NChinpi with donepezil and five patients receiving donepezil alone	NChinpi was prepared from 30 g/day of Citrus reticulata peel containing 0.44% nobiletin, administered as a decoction in three daily doses; 1 year.	One-year administration of a nobiletin-rich *Citrus reticulata* peel preparation was associated with less cognitive decline; a between-group difference was reported for the ADAS-J-cog score.	Very small sample; non-randomised design; no placebo; concomitant donepezil treatment; multicomponent peel preparation	Preliminary clinical evidence	[90]
Randomised, double-blind, placebo-controlled crossover trial	Adults aged ≥ 50 years with nocturia; 40 participants enrolled and 36 completed the study.	Mixture providing 30 mg nobiletin and 15 mg tangeretin once daily; 6 weeks per intervention, separated by a washout period of at least 2 weeks	A mixture providing 30 mg nobiletin and 15 mg tangeretin daily for 6 weeks did not improve the primary endpoint of nocturnal bladder capacity but modestly reduced nighttime voiding frequency.	Small sample; short intervention; combination with tangeretin; clinical outcome not directly related to ageing or geroprotection	Randomised clinical evidence	[91]

## Data Availability

No new data were created or analyzed in this study. Data sharing is not applicable to this article.

## Abbreviations

The following abbreviations are used in this manuscript:

3′-DMN	3′-demethylnobiletin
3′,4′-DMN	3′,4′-demethylnobiletin
3xTg-AD	triple-transgenic mouse model of Alzheimer’s disease
4′-DMN	4′-demethylnobiletin
5′-DMN	5′-demethylnobiletin
ABC	ATP-binding cassette
AD	Alzheimer’s disease
AKO	Antarctic krill oil
AKT	protein kinase B
ALD	alcohol-related liver disease
AMP	adenosine monophosphate
AMPK	AMP-activated protein kinase
AP-1	activator protein 1
ATP	adenosine triphosphate
A549	human lung adenocarcinoma epithelial cell line
Bax	Bcl-2-associated X protein
Bcl-2	B-cell lymphoma 2
BCRP	breast cancer resistance protein
BDNF	brain-derived neurotrophic factor
BMAL1	brain and muscle ARNT-like protein 1
C2C12	murine skeletal muscle myoblast cell line
CK	creatine kinase
CKD	chronic kidney disease
CLOCK	circadian locomotor output cycles kaput
COX-2	cyclooxygenase-2
Cry1–2	cryptochrome circadian regulator 1 and 2
CYP	cytochrome P450
CYP1A	cytochrome P450 family 1 subfamily A
CYP3A	cytochrome P450 family 3 subfamily A
DAMPs	damage-associated molecular patterns
DHA	docosahexaenoic acid
DMSO	dimethyl sulfoxide
DNA	deoxyribonucleic acid
EMT	epithelial–mesenchymal transition
ERK	extracellular signal-regulated kinase
GPx	glutathione peroxidase
IκB	inhibitor of nuclear factor kappa B
IL-1β	interleukin-1 beta
IL-6	interleukin-6
iNOS	inducible nitric oxide synthase
i.p.	intraperitoneal administration
JNK	c-Jun N-terminal kinase
LC3	microtubule-associated protein 1 light chain 3
LC3B	microtubule-associated protein 1 light chain 3 beta
LDH	lactate dehydrogenase
LDL	low-density lipoprotein
MAPK	mitogen-activated protein kinase
MDA	malondialdehyde
MDM2	mouse double minute 2 homolog
MFN1/2	mitofusin 1 and mitofusin 2
MIRO	mitochondrial Rho GTPase
miR-197	microRNA-197
mtDAMPs	mitochondrial damage-associated molecular patterns
mtDNA	mitochondrial DNA
NDP52	nuclear dot protein 52 kDa
NF-κB	nuclear factor kappa B
NNK	4-(methylnitrosamino)-1-(3-pyridyl)-1-butanone
NO	nitric oxide
NOB	nobiletin
Npas2	neuronal PAS domain protein 2
NRF1	nuclear respiratory factor 1
NSCLC	non-small cell lung cancer
OMM	outer mitochondrial membrane
OPTN	optineurin
PAMPs	pathogen-associated molecular patterns
PD-L1	programmed death-ligand 1
Per1–3	period circadian regulator 1–3
PGC-1α	peroxisome proliferator-activated receptor gamma coactivator 1-alpha
PGs	prostaglandins
P-gp	P-glycoprotein
PI3K	phosphoinositide 3-kinase
PINK1	PTEN-induced kinase 1
PMF	polymethoxylated flavone
PPARγ	peroxisome proliferator-activated receptor gamma
PRRs	pattern-recognition receptors
PSD	paradoxical sleep deprivation
PTEN	phosphatase and tensin homolog
p16INK4a	cyclin-dependent kinase inhibitor 2A (p16INK4a)
p21CIP1/WAF1	cyclin-dependent kinase inhibitor 1A
p38 MAPK	p38 mitogen-activated protein kinase
p53	tumour protein p53
p62/SQSTM1	sequestosome 1
p65/RelA	RELA proto-oncogene, NF-κB subunit
p50	nuclear factor NF-κB p50 subunit
RORα/RORγ	retinoic acid receptor-related orphan receptor alpha/gamma
ROS	reactive oxygen species
SA-β-gal	senescence-associated beta-galactosidase
SAMP8	senescence-accelerated mouse-prone 8
SASP	senescence-associated secretory phenotype
SIRT1	sirtuin 1
SMEDDS	self-microemulsifying drug delivery system(s)
SOD	superoxide dismutase
STAT3	signal transducer and activator of transcription 3
TFAM	mitochondrial transcription factor A
TGF-β	transforming growth factor beta
TLRs	Toll-like receptors
TNF-α	tumour necrosis factor alpha
VDAC	voltage-dependent anion channel
VCO_2_	carbon dioxide production
VO_2_	oxygen consumption
